# Effects of an App-Based Mindfulness Intervention during Pregnancy on the Infant’s Prenatal Androgen Exposure: A Randomized Controlled Pilot Trial

**DOI:** 10.3390/jcm12196142

**Published:** 2023-09-23

**Authors:** Eva-Maria Siegmann, Anna Eichler, Verena Nadine Buchholz, Jennifer Gerlach, Constanza A. Pontones, Adriana Titzmann, Nicolas Arnaud, IMAC-Mind Consortium, Christiane Mühle, Matthias W. Beckmann, Peter A. Fasching, Oliver Kratz, Gunther H. Moll, Johannes Kornhuber, Bernd Lenz

**Affiliations:** 1 Department of Psychiatry and Psychotherapy, Universitätsklinikum Erlangen, Friedrich-Alexander-Universität Erlangen-Nürnberg (FAU), 91054 Erlangen, Germany; 2 Department of Child and Adolescent Mental Health, Universitätsklinikum Erlangen, Friedrich-Alexander-Universität Erlangen-Nürnberg (FAU), 91054 Erlangen, Germany; 3 Department of Obstetrics and Gynecology, Universitätsklinikum Erlangen, Friedrich-Alexander-Universität Erlangen-Nürnberg (FAU), 91054 Erlangen, Germany; 4 German Centre for Addiction Research in Childhood and Adolescence, University Medical Centre Hamburg–Eppendorf, 20246 Hamburg, Germany; 5 Department of Addictive Behavior and Addiction Medicine, Central Institute of Mental Health (CIMH), Medical Faculty Mannheim, Heidelberg University, 68159 Mannheim, Germany

**Keywords:** mindfulness, digit ratio, prenatal programming

## Abstract

Prenatal androgen exposure modulates the development of the brain, with lasting effects on its function and behavior over the infant’s life span. Environmental factors during pregnancy, in particular maternal stress, have been shown to influence the androgen load of the unborn child. We here addressed the research gap on whether a mindfulness intervention or a pregnancy education administered to pregnant women more affects the androgen exposure of the unborn child (quantified by the proxies of second-to-fourth digit length ratio (2D:4D) and anogenital distance assessed one year after delivery and at delivery, respectively). Moreover, we tested the mindfulness intervention’s effects on maternal perceived stress, anxiety, depressiveness, and mindfulness. Pregnant women (gestation weeks 8–14) were randomized to a 15-week app-based mindfulness-oriented intervention (*N* = 72) or a pregnancy education intervention (control condition; *N* = 74). The mindfulness-oriented group did not significantly differ from the pregnancy education group in infants’ 2D:4D or anogenital distance (partial η^2^ ≤ 0.01) or in maternal stress, anxiety, depressiveness, or mindfulness. However, the descriptive results indicate that across pregnancy, stress and anxiety decreased and mindfulness increased in both groups. Overall, this study did not show that the mindfulness intervention (relative to the pregnancy education) reduced the prenatal androgen exposure of the unborn children or improved the maternal outcomes significantly.

## 1. Introduction

In animal experiments, prenatal androgen exposure has been shown to modify brain development with effects on brain structure, gene expression, and behavior that last into adulthood [1,2,3,4]. Supporting evidence from human studies demonstrates that fetal testosterone levels are associated with the activity of the brain reward system and with approach behavior [5] as well as with brain volumes [6] in later life. However, due to feasibility reasons and ethical constraints, direct measurements of prenatal androgen load in humans are limited, and experimental studies are not possible.

Therefore, researchers have identified proxies for prenatal androgen exposure, such as the second-to-fourth digit length ratio (2D:4D) and the anogenital distance. Lower 2D:4D is interpreted as a marker for higher intrauterine androgenization [7,8]. This assumption is supported by both animal experiments [9,10] and human evidence: males have lower 2D:4D than females [11]. Studies reported correlations between lower children’s 2D:4D and higher fetal amniotic testosterone/estradiol ratios [12] and between lower female newborns’ 2D:4D and higher testosterone in amniotic fluid [13]. Female twins with a male co-twin develop lower 2D:4D than female twins with a female co-twin [14,15]. Individuals with congenital adrenal hyperplasia have lower 2D:4D [16] and persons with Klinefelter syndrome [17,18] or androgen insensitivity syndrome [7,19] have higher 2D:4D. Based on observed sex differences, 2D:4D is thought to be established during the first trimester [20] with only small alterations afterward [21] but see also [22,23,24] and to be independent of activational sex hormone levels (i.e., direct effects) [25]. However, 2D:4D’s validity as a marker of prenatal androgen exposure has also been questioned [26,27,28]. Similar to 2D:4D, the anogenital distance has been suggested as a marker for prenatal androgen exposure [29,30] with higher values indicating a higher prenatal androgen load. The anogenital distance is subject to sexual dimorphism, with larger values in males relative to females [29]. Girls with congenital adrenal hyperplasia have been suggested to develop altered anogenital distance values [31], and an androgen receptor knock-out mouse model shows a shorter anogenital distance in male mice [32]. Hence, 2D:4D and anogenital distance are thought to give insight into prenatal androgen load and are used to investigate prenatal androgenization.

The two biomarkers have been related to human behavior and illnesses. Lower 2D:4D has been linked to aggression [33], attention and behavioral problems [34,35], autism spectrum disorder [36], lower emotional stability [37], substance and non-substance-related addictive behaviors [38,39,40,41,42,43,44], suicidality [45], and lower life expectancy [46]. It has also been associated with transgender identity [47] and brain volumes [23,48]. These findings are often influenced by sex. Anogenital distance at birth is related to masculine play behavior in boys three to four years of age [29]. Moreover, previous studies have related the 2D:4D and anogenital distance of offspring to maternal behaviors during pregnancy. Maternal stress, alcohol consumption, and cigarette smoking during pregnancy were associated with lower 2D:4D in the children [44,49]. Similarly, in rodents, higher maternal corticosterone during pregnancy was related to lower 2D:4D in the offspring [50]. Moreover, prenatal exposure to stressful life events [51,52] and maternal smoking [53,54,55] have been associated with masculinized anogenital distance in humans, with evidence for a moderating role of sex. Thus, maternal stress and the related prenatal androgen exposures might evolve as a potential target to improve the offspring’s later health [56].

Symptoms of psychological distress are often co-occurring during pregnancy [57] with approximately 20% of expectant mothers reporting depressive [58] and/or anxiety symptoms [59] as well as 50% of pregnant women experiencing significant prenatal stress [60]. A mother’s high psychological burden influences the child’s development, entailing, for example, a higher risk of premature birth [61] and a lower birth weight [62], but also implicates difficulties in the child’s later life such as neurodevelopmental impairments, behavioral disturbances, and psychosocial difficulties [63,64,65,66].

Treatment and prevention programs for pregnant women are subject to scientific research with promising results for interpersonal therapy (IPT, [67]), psychoeducational programs [68], cognitive behavioral therapy (CBT, [69,70]), and acceptance and commitment therapy (ACT, [71,72]). Recent systematic reviews and meta-analyses suggest that mindfulness-based interventions are effective or even superior to other forms of treatment in reducing psychological distress among expectant mothers [73,74,75]. Mindfulness-oriented treatments can be characterized by cultivating a non-judgmental and non-reactive awareness as well as an acceptance of inner thoughts, feelings, and body sensations in the present moment [76,77]. 

As in the case of all face-to-face treatment programs, there are potential structural barriers when administering a mindfulness-based intervention in person, for example, a lack of professionals, high costs, geographic remoteness, and a long waiting time [73,74]. These barriers can be circumvented by developing a digital mindfulness-based intervention, which can be easily accessible and more cost-effective once established. While the promising effect of digital, internet-based, or e-health mindfulness interventions on reducing psychological symptoms in pregnant women has consistently been shown [73,74,75], studies on the interventions’ influence on the children’s health or on mediators are rare [78,79,80] or lacking regarding the prenatal androgen load.

### Aims of the Study

We here addressed this research gap and investigated whether an app-based mindfulness-oriented intervention versus a pregnancy education intervention administered to pregnant women affects the androgen exposure of the unborn child (using 2D:4D and anogenital distance as proxies). We also tested the mindfulness intervention’s effects on maternal perceived stress, anxiety, depressiveness, and mindfulness.

## 2. Materials and Methods

### 2.1. Study Cohort and Intervention

For this monocentric, prospective, controlled, and investigator-blinded trial, 207 pregnant women were screened at the Department of Obstetrics and Gynecology of the University Hospital Erlangen. Inclusion criteria were 8 + 0 to 14 + 0 weeks of pregnancy, 18 to 50 years of age, and unproblematic pregnancy at the time of inclusion. All women provided written informed consent. Women with multiple pregnancies, problems understanding the introductory briefing, prior or current severe psychiatric disorders, or a history of adverse or missing effects of mindfulness exercises were excluded. Following our a priori sample size calculation (see the published study protocol [56]), we intended to investigate a per protocol sample of 260 study participants (Cohen’s d = 0.35 for the primary endpoint, significance level of 0.05, power of 0.80). Physicians of the Department of Obstetrics and Gynecology of the University Hospital Erlangen enrolled and assigned participants to the study groups. The Ethics Committee of the Medical Faculty of the Friedrich-Alexander-Universität Erlangen-Nürnberg (FAU) approved the study protocol (application number: 58_18 B). The study was pre-registered in the German Register of Clinical Trials (DRKS00014920). For further details of the study design, see [56]. We here focused on the primary endpoint infant 2D:4D as well as the infant anogenital distance and maternal stress, depressiveness, anxiety, and mindfulness.

The women were randomized 1:1 to participate in either a 15-week app-based mindfulness-oriented intervention or a pregnancy education intervention (stratification: nullipara versus non-nullipara, high versus low levels of stress and mindfulness according to the Perceived Stress Scale (PSS-10, [81]) and the Mindful Attention and Awareness Scale (MAAS, [82]) scores, respectively). The random allocation sequence was generated with the program secuTrial (https://www.secutrial.com/, accessed on 2 February 2018) following the above-described rules of stratification and was completely concealed from the assigning physician. In the mindfulness-oriented intervention group, the women received mindfulness exercises via audio recording twice per week. The topics addressed included mindfulness in everyday life, distinguishing emotions and thoughts, not losing oneself in thoughts, and mindful breathing to remain in the present moment. We instructed the participants to exercise up to seven times per week. In the pregnancy education intervention group, the women were provided with audio recordings twice per week on general information on pregnancy, delivery, and breast-feeding. Both groups attended three onsite study visits (baseline (V1), day 53 ± 7 (V15), and day 105 ± 7 (V29)) during pregnancy as well as a delivery visit (delivery + up to 14 days (V30)) and an 11–12 months postpartum visit (V31)). For further details of the study design, see [56]. Visits V15 and V29 were converted to telephone contacts during the COVID-19 pandemic. During visits V30 and V31, the raters were blinded to the participants’ group allocation. 

### 2.2. Markers of Prenatal Androgen Exposure

#### 2.2.1. Second-to-Fourth Digit Length Ratio (2D:4D)

During the 11–12 months postpartum visit, we scanned the children’s and mothers’ palms of the right and left hands with an HP Scanjet G4050 in gray level with 300 DPI resolution. The GNU Image Manipulation Program (GIMP; www.gimp.org) was used to measure the lengths of the second (2D) and fourth (4D) digits, which was defined as the distance between the middle of the basal crease and the digits’ tips. Each digit was measured three times by three independent raters (i.e., nine times). The raters were blinded to the group allocation and the infants’ sex. The average of right hand and left hand 2D:4D (A2D:4D) was analyzed as primary endpoint, and right hand 2D:4D (R2D:4D) and left hand 2D:4D (L2D:4D) as further endpoints. We found high interrater reliabilities (two-way random effects intraclass correlation coefficient (ICC), absolute agreement, mean of three raters with confidence interval (CI)) for the children’s digit ratios: (1) A2D:4D: ICC = 0.99 (95% CI [0.98; 0.99]); (2) R2D:4D: ICC = 0.98 (95% CI [0.98; 0.99]); (3) L2D:4D: ICC = 0.98 (95% CI [0.97; 0.98]). 

#### 2.2.2. Anogenital Distance

During the delivery study visit (V30), we measured the anoscrotal distance in boys (center of the anus to the most posterior, midline point of the perineoscrotal junction) and the anofourchettal distance in girls (center of the anus to the posterior convergence of the fourchette) as described by Thankamony et al. [29] because these measures are commonly used in epidemiological studies.

### 2.3. Maternal Behavioral Phenotyping

At baseline (V1), during (interim V15 and V29), and after the intervention (delivery V30, 11–12 months postpartum V31), we measured the mothers’ self-reported psychological distress operationalized via their stress level (PSS-10), their pregnancy-related anxiety symptoms (Pregnancy-Related Anxiety Questionnaire—Revised, PRAQ-R2), and their depressive symptoms (Edinburgh Postnatal Depression Scale, EPDS) and we evaluated the effectiveness of our mindfulness-oriented intervention by assessing the mothers’ mindfulness levels with the German versions of the self-report questionnaires MAAS at V1, V15, V29, V30, and V31 and the Five Facet Mindfulness Questionnaire (FFMQ-D) at V1, V15, and V29 [81,82,83,84,85,86].

### 2.4. Statistical Analyses

We present the data as means and standard deviations or relative frequencies. The χ^2^ test was applied to assess differences in nominal, ordinal, and non-normally distributed variables (e.g., marital status, employment status, previous births). Continuous variables were compared using t-tests (e.g., 2D:4D, anogenital distance, body mass index). The data were analyzed using the statistical software R, version 4.1.3 [87]. *p* < 0.05 (two-sided) was set as level of significance.

#### 2.4.1. Markers of Prenatal Androgen Exposure

Group differences in the endpoints children’s A2D:4D, R2D:4D, and L2D:4D were tested with analyses of covariance (ANCOVA) with infants’ 2D:4D as dependent variable, group allocation (mindfulness versus pregnancy education) as independent variable and the covariates infant’s sex, age at follow-up, and mother’s 2D:4D with their potential influence on the dependent variable. The infant’s sex needs to be controlled for since sex differences between male and female children were to be expected [13,24]. Moreover, studies suggest differences in 2D:4D depending on children’s age [23,24,27] and associations between the mothers’ and children’s 2D:4D are reported, particularly in girls [13,24]. Group differences in the children’s anogenital distance were again assessed via ANCOVA, with anogenital distance as dependent variable, group allocation (mindfulness versus pregnancy education) as independent variable, and the children’s sex and gestational age at birth as covariates. We report the estimated marginal means with corresponding 95% confidence intervals. Effect sizes are reported as partial η^2^.

#### 2.4.2. Maternal Behavioral Phenotyping

We evaluated the effectiveness of the mindfulness intervention by comparing the groups of mothers (mindfulness versus pregnancy education) in terms of stress, anxiety, depressiveness, and mindfulness across pregnancy (see Section 2.3. Maternal Behavioral Phenotyping). Stress (PSS-10), depressiveness (EPDS), and mindfulness (MAAS, FFMQ-D) were separately analyzed using mixed linear models with treatment group as fixed effect, participant as random effect, and baseline questionnaire score as covariate. We report model-based group differences (adjusted for baseline score) with corresponding 95% confidence intervals. Mixed models provide unbiased estimation if the missing at-random assumption holds. Regarding anxiety, we computed the difference in the PRAQ-R2 score between V29 and V1 and compared it between the mindfulness and the pregnancy education groups using an ANCOVA corrected for baseline PRAQ-R2 scores.

#### 2.4.3. Dropout Analyses

We performed dropout analyses to identify potential systematic discontinuation among our participants. Spearman rank correlations, or phi coefficients, were computed between dropout (yes versus no) and relevant baseline characteristics, which are further described in Appendix A. In a second step, we performed logistic regression analyses with dropout (yes versus no) as outcome and those baseline variables that showed significant correlations (*p* < 0.05) or trends towards a significant association (*p* < 0.10) as independent variables (see Appendix A, Table A1).

## 3. Results

### 3.1. Sociodemographic Characteristics

**Figure 1:**
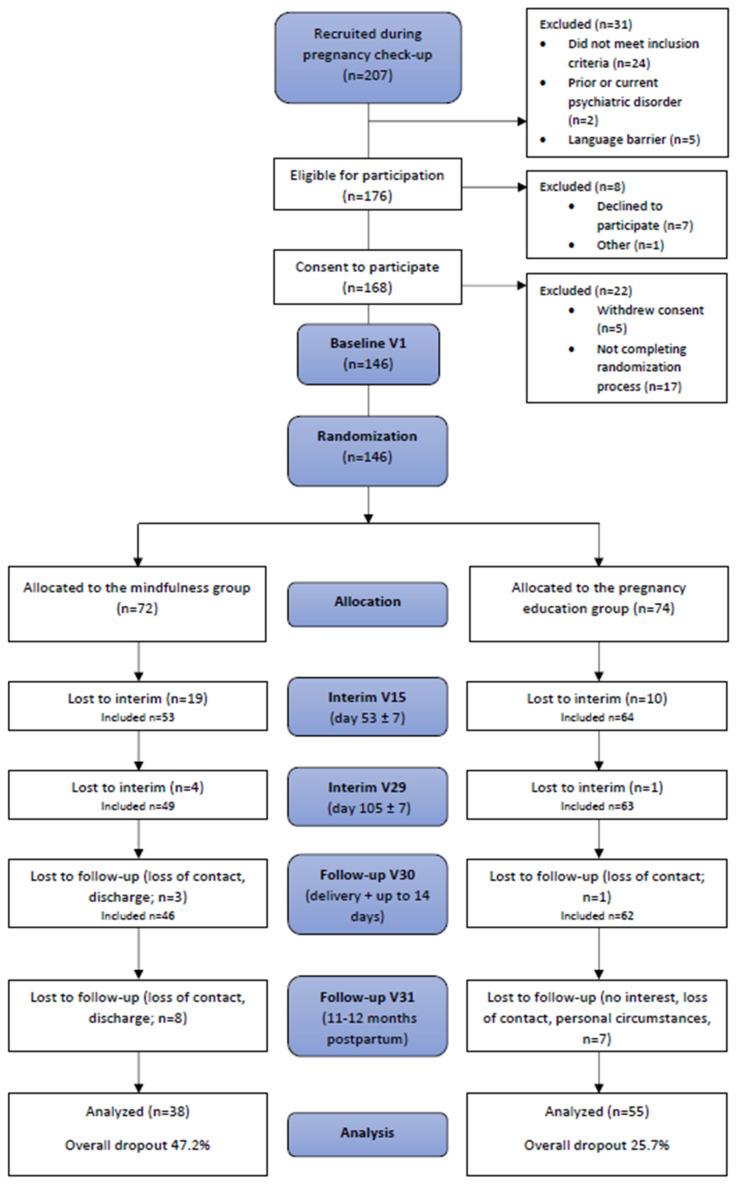
The participant flowchart illustrating recruitment, randomization, and dropout processes. The number of participants analyzed includes every participant contributing any outcome of interest at follow-up V31.

We screened 207 pregnant women during their routine pregnancy check-ups from February 2020 to February 2022. The baseline recruiting phase was stopped in February 2022 in order to ensure that every participant could complete the last study visit, V31, 11–12 months post-partum before the study duration expires. Prior to randomization, we excluded 61 women (for reasons of exclusion, see Figure 1) and randomized 146 participants, *n* = 72 participants in the mindfulness group and *n* = 74 participants in the pregnancy education group. Ultimately, 93 mothers and their infants (38 in the mindfulness group, 55 in the pregnancy education group) completed the study and were included in the analysis (see Figure 1), corresponding to a significantly higher dropout rate of 47.2% in the mindfulness group than in the pregnancy education group of 25.7%. Detailed dropout analyses can be found in Appendix A and Table A1. In July 2023, the last study subject was examined. As shown in Table 1, 45.7% of infants were of male sex. The demographic and clinical characteristics were not significantly different between the groups (Table 1). The 2D:4D values were obtained from 76 mothers and 68 children.

### 3.2. Intervention Effects on Second-to-Fourth Digit Length Ratio (2D:4D) and Anogenital Distance

We did not detect significant differences in the children’s 2D:4D between the mindfulness group and the pregnancy education group (ANCOVAs; A2D:4D: *F*(1,43) = 0.18, *p* = 0.672; R2D:4D: *F*(1,56) = 0.63, *p* = 0.431; L2D:4D: *F*(1,53) = 0.01, *p* = 0.905). The covariate children’s sex was not significantly associated with the children’s 2D:4D (A2D:4D: *F*(1,43) = 0.004, *p* = 0.950; R2D:4D: *F*(1,56) = 1.23, *p* = 0.272; L2D:4D: *F*(1,53) = 0.31, *p* = 0.582), whereas the covariate mother’s 2D:4D showed a significant link (A2D:4D: *F*(1,43) = 7.15, *p* = 0.011; R2D:4D: *F*(1,56) = 9.17, *p* = 0.004; L2D:4D: *F*(1,53) = 6.27, *p* = 0.015). The infants’ age at follow-up revealed a significant association with R2D:4D (*F*(1,56) = 6.57, *p* = 0.013), but not with A2D:4D (*F*(1,43) = 0.30, *p* = 0.584) or L2D:4D (*F*(1,53) = 0.15, *p* = 0.698). Corresponding effect sizes are displayed in Table 2. Regarding A2D:4D, the estimated marginal means (with age at follow-up = 11.60 months and mother’s 2D:4D = 0.969) are 0.923 (95% CI [0.909; 0.938]) in the mindfulness group and 0.926 (95% CI [0.913; 0.938]) in the pregnancy education group. The estimated marginal means for R2D:4D (with age at follow-up = 11.70 months and mother’s 2D:4D = 0.968) are 0.917 (95% CI [0.901; 0.933]) in the mindfulness group and 0.924 (95% CI [0.911; 0.937]) in the pregnancy education group. For L2D:4D, the estimated marginal means (with age at follow-up = 11.70 months and mother’s 2D:4D = 0.971) are 0.922 (95% CI [0.906; 0.938]) in the mindfulness group and 0.922 (95% CI [0.906; 0.937]) in the pregnancy education group.

The ANCOVA comparing the infants’ anogenital distance between the mindfulness and pregnancy education groups revealed no significant effect of the group (*F*(1,57) = 2.108, *p* = 0.152, partial η^2^ = 0.012) or the covariate gestational age at birth (*F*(1,57) = 0.056, *p* = 0.814, partial η^2^ = 0.001), but yielded significant results for the covariate infant’s sex (*F*(1,57) = 44.215, *p* < 0.001, partial η^2^ = 0.431). Boys had a significantly greater anogenital distance (M ± SD, 2.734 cm ± 0.790) than girls (M ± SD, 1.715 cm ± 0.630). The estimated marginal means (with gestational age at birth = 39.0 weeks) are 2.34 cm (95% CI [2.00; 2.68]) in the mindfulness group and 2.12 cm (95% CI [1.80; 2.44]) in the pregnancy education group.

### 3.3. Intervention Effects on the Mothers’ Stress, Anxiety, Depressiveness, and Mindfulness Levels

In Appendix B, Table A2, the adjusted means and the adjusted differences are displayed for each mixed linear model (stress, depressiveness, and mindfulness).

#### 3.3.1. Stress Scores (PSS-10)

The mixed linear model did not reveal a significant effect of the group (mindfulness versus pregnancy education) (χ^2^(1) = 1.202, *p* = 0.273), but did for measurement time point (χ^2^(5) = 12.553, *p* = 0.028) and baseline PSS-10 scores (χ^2^(1) = 64.522, *p* < 0.001). Adjusted means (corrected for baseline PSS-10 score) for each measurement time point (see Table A2) indicate that stress levels decreased from study inclusion to follow-up (V31) without a meaningful difference between the two intervention groups.

#### 3.3.2. Anxiety Scores (PRAQ-R2)

The ANCOVA did not reveal a significant difference in the change in PRAQ-R2 score between the mindfulness and pregnancy education groups (*F*(1) = 2.11, *p* = 0.150) nor a significant impact of baseline PRAQ-R2 score (*F*(1) = 2.03, *p* = 0.158). The estimated marginal means (with baseline PRAQ-R2 score = 23.0) are −1.84 (95% CI [−3.42; −0.26]) in the mindfulness group and −0.25 (95% CI [−1.70; 1.20]) in the pregnancy education group, indicating a descriptively larger reduction in anxiety levels for the mindfulness group.

#### 3.3.3. Depressiveness Scores (EPDS)

The mixed linear model did not reveal a significant effect of the group (mindfulness versus pregnancy education) (χ^2^(1) = 0.001, *p* = 0.970) or measurement time point (χ^2^(3) = 1.607, *p* = 0.658), but did for baseline EPDS scores (χ^2^(1) = 70.574, *p* < 0.001). Adjusted means (corrected for baseline EPDS score) for each measurement time point (see Table A2) suggest that descriptive depressiveness scores first increased, then decreased approaching birth, and increased again at follow-up one year after birth (V31).

#### 3.3.4. Mindfulness Scores (MAAS)

The mixed linear model did not reveal a significant effect of the group (mindfulness versus pregnancy education) (χ^2^(1) = 1.647, *p* = 0.199) or measurement time point (χ^2^(5) = 7.380, *p* = 0.194), but did for baseline MAAS scores (χ^2^(1) = 162.052, *p* < 0.001). Adjusted means (corrected for baseline MAAS score) for each measurement time point (see Table A2) indicate that trait mindfulness increased continuously from study inclusion to follow-up (V31) to a similar extent in both groups.

#### 3.3.5. Mindfulness Scores (FFMQ-D)

The mixed linear model did not reveal a significant effect of the group (mindfulness versus pregnancy education) (χ^2^(1) = 1.199, *p* = 0.274) or measurement time point (χ^2^(1) = 1.819, *p* = 0.177), but did for baseline FFMQ-D scores (χ^2^(1) = 180.620, *p* < 0.001). Adjusted means (corrected for baseline FFMQ-D score) for each measurement time point (see Table A2) suggest that trait mindfulness increased continuously from study inclusion to interim V29 to a similar extent in both groups.

## 4. Discussion

In this trial, we administered an app-based mindfulness-oriented intervention to pregnant women and tested its effects on the children’s prenatal androgen load (assessed via 2D:4D and anogenital distance) and the mothers’ stress, anxiety, depressiveness, and mindfulness levels against a pregnancy education intervention. However, we did not find evidence for significant differences between the two groups.

In our a priori sample size calculation (see the published study protocol [56]), we expected a standardized group difference in Cohen’s d = 0.35 for the primary endpoint A2D:4D. Using a significance level of 0.05 and aiming at a power of 0.80, we intended to investigate a per protocol sample of 260 study participants. Mainly due to the COVID-19 pandemic, we were not able to reach this goal and tested the A2D:4D values of 22 children in the mindfulness group against the A2D:4D values of 30 children in the pregnancy education group, resulting in an achieved power of only 7% (partial η^2^ = 0.003, α = 0.05, two groups, three covariates). We also did not detect significant sex differences between male and female subjects, which is usually a common finding in 2D:4D research [11]. This might also be due to the low power achieved in our study. Although we missed the preplanned sample size by far, the variances in the 2D:4D and anogenital distance explained by the group condition are negligible with partial η^2^ ≤ 0.01. We are thus tempted to speculate that we would also have detected no significant effects even if we had reached the a priori calculated sample size. Nevertheless, the null findings of this study must certainly be interpreted as preliminary, and the project needs to be seen as a pilot trial.

The results indicate that across pregnancy, stress and anxiety decreased and mindfulness increased in the mindfulness group, but also in the pregnancy education group. We failed to find evidence for the superior effect of the mindfulness intervention. It is possible that most women are regularly informed about and also trained in mindfulness within the frame of routine pregnancy care, which is supported by this study’s observation of increasing mindfulness across pregnancy in both groups. An example might be birthing classes, which are very common in Germany; women receive general information on pregnancy and health, but also on mindful breathing during delivery or on mindful observation of bodily sensations during pregnancy, which are both concepts inherent to typical mindfulness intervention definitions [76,77]. Additionally, mothers are thus encouraged to pay more attention to themselves, which influences and potentially biases their self-report in questionnaires. We also speculate that pregnancy education per se might be able to reduce stress and anxiety since it reduces insecurities. This assumption agrees with a very recently published study of 229 women, showing that an online mindfulness-based intervention does not significantly excel care as usual in terms of pregnancy distress [88]. Moreover, it is possible that the COVID-19 pandemic has affected the intervention effects, and it is unclear whether the effects observed here can be generalized. Overall, our sample does not seem to be overly burdened with psychological distress (see PSS-10 [81], PRAQ-R2 [83], and EPDS scores [84,85] in Table 1 and Table A2). The administration of our mindfulness intervention to a population with a higher symptom load, e.g., psychiatric patients or particularly stressed mothers, might yield more beneficial effects. 

As the mindfulness and pregnancy education groups did not differ in stress, anxiety, or depressiveness, this study cannot answer whether interventional reductions in these burdens are able to influence prenatal androgen exposures and related long-term consequences on the children’s behavior and illness risks. This investigation also does not allow for concluding whether 2D:4D and the anogenital distance are valid assessment tools for the prenatal androgen load among infants. Recent meta-analyses show that app- or internet-based interventions are less effective than human-guided or face-to-face interventions, particularly in terms of commitment to study completion [89,90]. This might be owing to less personal interaction and, thus, less perceived social support [90]. Our study was also subject to a high dropout rate. Only the A2D:4D of 52 children from the 146 included pregnant women could be analyzed here. We expected that direct interventions would result in lower dropout rates. The significantly higher dropout rate in the mindfulness group compared to the pregnancy education group observed in this study might suggest that the mindfulness intervention was not sufficiently attractive due to the conversion of visits during the COVID-19 pandemic into the less personal telephone contact or target groups tailored to pregnant women. Thus, future studies are advised to use more established stress reduction therapies, to compare their effects on the prenatal androgen load with those of less active control groups, such as waiting list groups, and to investigate the effectiveness of the intervention in a sample with a higher symptom load. Assessment techniques other than self-report questionnaires are also advisable, as well as blood samples to quantify direct hormonal influences and motivating measures to increase intervention adherence. Although we aimed at including mothers at 8 + 0 to 14 + 0 weeks of pregnancy, the actual gestational age at enrollment was rather late (see Table 1), which might limit the interventional effects (see Section 4.1). We advise future studies to ensure an earlier gestational age at enrollment. In addition, there are difficulties in defining mindfulness [91], and the open question remains of which factors moderate androgenic actions, which both require future research.

### 4.1. Strengths and Limitations

The strengths of this trial include the preregistration and the prospective, controlled, and investigator-blinded study design. Emphasis needs to be laid on the fact that this investigation examines the impact of parental intervention on different children’s developmental outcomes, which is rarely addressed in mindfulness research. Furthermore, it allows insights into the psychological well-being of pregnant women during the exceptional situation of the COVID-19 pandemic. The investigation is mainly limited by the low number of participants included and analyzed, which led to its conversion to a pilot randomized controlled trial. Additionally, legal contact constraints entailed by the COVID-19 pandemic caused a change in the study conduct during the recruitment phase. We also note that the improvements over time in mothers’ psychological well-being (see Table A2) are very small and not in the range of clinically relevant effects for both groups. Moreover, the reliability and validity of the 2D:4D and anogenital distance as proxies for the prenatal androgen load have been criticized, especially during early life [26,27,29]. We did not validate the proxies with direct hormonal measurements. As sexual differentiation of the fingers is thought to be established during the first trimester [20], our intervention—with a mean gestational age at enrollment of 12.5 weeks (see Table 1)—is rather late to develop full effects. 

## 5. Conclusions

Overall, this study did not show that the app-based mindfulness intervention (relative to the pregnancy education intervention) administered to pregnant women mainly during the COVID pandemic time reduced the prenatal androgen exposure of the unborn children or influenced the maternal stress, anxiety, depressiveness, or mindfulness levels.

## Figures and Tables

**Table 1:** Sociodemographic characteristics of all included participants.

	Mindfulness Group (*n* = 72)		Pregnancy Education Group (*n* = 74)			
	*N*	M ± SD (%)	*N*	M ± SD (%)	t, χ^2^	*p*
Maternal age (years)	71	32.77 ± 4.39	74	32.73 ± 4.88	0.1	0.954
Marital status					5.6	0.127
Single	19	27.9%	20	30.3%		
Married	47	69.1%	38	57.6%		
Other	2	2.9%	8	12.1%		
Maternal education					0.0	1.0
>12 years	30	44.1%	29	43.9%		
≤12 years	38	55.9%	37	56.1%		
Employment status at baseline					5.4	0.124
Working (full/part-time)	58	85.3%	54	83.1%		
Not working	10	14.7%	11	16.9%		
Health insurance					0.0	1.0
Public	60	88.2%	58	89.2%		
Private/other	8	11.8%	7	10.8%		
BMI (kg/m^2^)	72	23.93 ± 4.32	74	24.38 ± 3.91	0.9	0.342
Has previously practiced meditation					2.4	0.123
Yes	30	44.1%	40	58.8%		
No	38	55.9%	28	41.2%		
Previous births					1.7	0.189
0	36	50.0%	46	62.2%		
1	36	50.0%	28	37.8%		
Gestational age at enrollment (weeks)	66	12.65 ± 1.48	61	12.56 ± 1.91	0.3	0.763
Gestational age at birth (weeks)	42	39.19 ± 1.57	50	38.82 ± 2.05	1.0	0.340
Delivery mode					0.8	0.364
Vaginal	27	61.4%	41	71.9%		
Caesarian	17	38.6%	16	28.1%		
PSS-10 score ^a^	72	15.97 ± 5.77	74	15.70 ± 5.43	0.3	0.772
PRAQ-R2 score ^a^	66	23.77 ± 6.81	66	21.94 ± 5.48	2.0	0.160
EPDS score ^a^	65	7.88 ± 5.12	65	6.71 ± 5.09	2.2	0.140
FFMQ-D score ^a^	65	133.05 ± 17.27	58	135.41 ± 16.01	−0.8	0.434
MAAS score ^a^	72	4.17 ± 0.80	74	4.24 ± 0.68	−0.6	0.539
Infant’s sex					0.0	0.951
Male	23	50.0%	28	47.5%		
Female	23	50.0%	31	52.5%		
Infant’s anogenital distance (cm)	33	2.28 ± 0.83	40	2.13 ± 0.91	0.7	0.460
Mothers’ A2D:4D	32	0.97 ± 0.03	43	0.97 ± 0.03	0.3	0.742
Mothers’ R2D:4D	32	0.97 ± 0.03	44	0.97 ± 0.03	0.0	0.995
Mothers’ L2D:4D	32	0.97 ± 0.03	43	0.97 ± 0.03	0.4	0.681
Infants’ A2D:4D	22	0.92 ± 0.04	30	0.93 ± 0.03	−0.4	0.689
Infants’ R2D:4D	26	0.92 ± 0.04	39	0.93 ± 0.04	−0.9	0.373
Infants’ L2D:4D	29	0.92 ± 0.04	33	0.92 ± 0.05	0.1	0.888

^a^ Assessed at baseline V1. A2D:4D, average second-to-fourth digit length ratio of the right and left hands; BMI, body mass index; EPDS, Edinburgh Postnatal Depression Scale; FFMQ-D, Five Facet Mindfulness Questionnaire; L2D:4D, second-to-fourth digit length ratio of the left hand; MAAS, mindful attention and awareness scale; PRAQ-R2, pregnancy-related anxiety questionnaire—revised; PSS-10, perceived stress scale; R2D:4D, second-to-fourth digit length ratio of the right hand.

**Table 2:** Effect sizes of the ANCOVAs with the second-to-fourth finger length ratios (2D:4D) as dependent variables (partial η^2^).

	Group (Mindfulness versus Pregnancy Education)	Infant’s Sex	Age at Follow-Up	Mother’s 2D:4D
A2D:4D	0.003	0.001	0.005	0.141
R2D:4D	0.009	0.013	0.068	0.128
L2D:4D	0.00004	0.003	0.006	0.105

Abbreviations: A2D:4D, average 2D:4D of the right and left hands; R2D:4D, 2D:4D of the right hand; L2D:4D, 2D:4D of the left hand.

## Data Availability

The data presented are available upon request from the corresponding author.

## Appendix A

### Dropout Analyses

In the first step, we analyzed the association between data availability at follow-up (V31) and different potential confounders: (1) Participant characteristics, namely group allocation (mindfulness versus pregnancy education), mothers’ age at enrollment, previous experiences with meditation and relaxation training (yes versus no), smoking status (smoker versus non-smoker), alcohol consumption (yes versus no), migration background (born in Germany: yes versus no), body mass index, marital status (single versus married versus other), and level of education (>12 years versus ≤12 years). (2) Pregnancy-related variables, namely, number of previous miscarriages, gestational age at enrollment (in weeks), number of previous births, and joy regarding the current pregnancy (from 0 = “not at all” to 4 = “a lot”). (3) Baseline scores of self-report variables, namely stress level (PSS-10 and all subscales), pregnancy-related anxiety (PRAQ-R2 and all subscales), depressiveness (EPDS), and trait mindfulness (MAAS, FFMQ-D, and all subscales). All of these variables were collected as part of the study, but they are not all subjects of the analyses in this paper. For more detailed information on applied measures, see the study protocol [56]. We found a significant association between data availability at V31 and the variable group allocation (phi coefficient = −0.22, *p* = 0.011), suggesting a higher drop-out in the mindfulness versus the pregnancy education group. Moreover, we found a trend towards a significant association between data availability at V31 and previous experiences with meditation and relaxation strategies (phi coefficient = −0.16, *p* = 0.089), indicating that less experience might lead to a higher risk of drop-out. In the second step, logistic regressions were used to predict data availability at V31 from group allocation and previous experience. The full model with both variables (model 1) showed a meaningful effect for group allocation (OR = 0.33, 95% CI [0.15; 0.70]) and no meaningful effect for previous experience (OR = 0.57, 95% CI [0.26; 1.12]). This means that the chance of dropping out of the study was 3.03 times higher for participants in the mindfulness group compared to the pregnancy education group. By omitting the non-significant predictor, a more parsimonious model with group allocation only (model 2) was created. Model 2 revealed a meaningful effect for group allocation (OR = 0.39, 95% CI [0.19; 0.77]), but a worse model fit with Nagelkerke’s R^2^ = 0.06 (see Table A1).

**Table A1:** Logistic regression predicting availability of data at follow-up V31.

Predicted Outcome	Predictor(s)	OR	95% CI	*p*	Model Parameters
Model 1					
Data at V31 available	Group allocation Previous experience	0.33 0.57	0.15–0.70 0.26–1.12	0.005 0.137	χ^2^ = 12.01, df = 2, *p* = 0.002 Nagelkerke’s R^2^ = 0.11
Model 2					
Data at V31 available	Group allocation	0.39	0.19–0.77	0.007	χ^2^ = 7.40, df = 1, *p* = 0.006 Nagelkerke’s R^2^ = 0.06

V31, 11–12 months postpartum visit.

## Appendix B

**Table A2:** Adjusted means and adjusted differences for all mixed linear models.

Questionnaire	Measurement Timepoint	Mindfulness Group		Pregnancy Education Group		
		*N*	Adjusted means (95% CI)	*N*	Adjusted means (95% CI)	Adjusted Difference (95% CI)
PSS-10						−0.87 (−2.43; 0.70)
	V15	46	13.60 (12.25; 15.00)	51	12.80 (11.44; 14.10)	
	V29	42	13.70 (12.31; 15.10)	50	12.80 (11.51; 14.20)	
	V30	38	12.90 (11.45; 14.30)	46	12.00 (10.66; 13.40)	
	V31	33	12.10 (10.68; 13.60)	47	11.30 (9.92; 12.60)	
EPDS						−0.02 (−1.11; 1.06)
	V15	47	5.58 (4.65; 6.52)	59	5.56 (4.70; 6.42)	
	V29	45	5.68 (4.74; 6.63)	56	5.66 (4.79; 6.53)	
	V30	37	5.15 (4.17; 6.14)	52	5.13 (4.23; 6.03)	
	V31	35	5.52 (4.52; 6.51)	52	5.50 (4.60; 6.40)	
FFMQ-D						−2.52 (−7.13; 2.08)
	V15	8	138.00 (132.00; 144.00)	15	136.00 (130.00; 141.00)	
	V29	41	141.00 (138.00; 145.00)	49	139.00 (136.00; 142.00)	
MAAS						0.12 (−0.07; 0.31)
	V15	45	4.26 (4.10; 4.42)	51	4.38 (4.23; 4.54)	
	V29	42	4.37 (4.20; 4.53)	50	4.49 (4.33; 4.65)	
	V30	37	4.39 (4.22; 4.56)	44	4.51 (4.35; 4.68)	
	V31	34	4.41 (4.24; 4.58)	50	5.53 (4.37; 4.69)	

Abbreviations: EPDS, Edinburgh Postnatal Depression Scale; FFMQ-D, Five Facet Mindfulness Questionnaire; MAAS, mindful attention and awareness scale, PSS-10, perceived stress scale; V15, day 53 ± 7 after enrollment; V29, day 105 ± 7 after enrollment; V30, delivery + up to 14 days; V31, 11–12 months postpartum.
